# Lack of Detection of *Toxoplasma gondii* in *Pipistrellus* spp. Bats from Densely Cat-Populated Areas of NE Spain

**DOI:** 10.3390/pathogens11121451

**Published:** 2022-12-01

**Authors:** Lourdes Lobato-Bailón, Ane López-Morales, Rita Quintela, Maria Puig Ribas, Rafael Molina-López, Elena Obon, Sebastián Napp, Lola Pailler-García, Johan Espunyes, Óscar Cabezón

**Affiliations:** 1 Wildlife Conservation Medicine Research Group (WildCoM), Departament de Medicina i Cirurgia Animals, Universitat Autònoma de Barcelona, 08193 Bellaterra, Spain; 2 Centre de Fauna Salvatge de Torreferrussa, Direcció General del Medi Natural-Forestal Catalana, SA, Generalitat de Catalunya, 08130 Santa Perpètua de la Mogoda, Spain; 3 Unitat Mixta d’Investigació IRTA-UAB en Sanitat Animal, Centre de Recerca en Sanitat Animal (CReSA), Campus de la Universitat Autònoma de Barcelona, 08193 Bellaterra, Spain

**Keywords:** *Chiroptera*, *Vespertilionidae*, wildlife, diseases, host–pathogen interactions, health, *Sarcocystidae*, *Toxoplasma gondii*

## Abstract

*Toxoplasma gondii* infection in healthy animals is often asymptomatic. However, some species with little history of contact with the parasite, such as marsupials and New World primates, present high mortality rates after infection. Despite its potential conservation concern, *T. gondii* infection in insectivorous bats has received little attention, and its impact on bat populations’ health is unknown. To assess the putative role of insectivorous bats in the cycle of *T. gondii*, samples of three species of bats (*Pipistrellus pipistrellus*, *P. pygmaeus* and *P. kuhlii*) collected between 2019 and 2021 in NE Spain were tested for the presence of the parasite using a qPCR. All tissues resulted negative (0.0% prevalence with 95% CI: [0.0–2.6]) for the presence of *T. gondii*. Unlike previous studies on insectivorous bats from Europe, Asia and America, the present study suggests that *Pipistrellus* spp. bats do not play a significant role in the epidemiology of *T. gondii* in NE Spain. Further studies are encouraged to elucidate both the epidemiology of *T. gondii* and its potential impact on the health of microchiropteran species in Europe.

## 1. Introduction

The order *Chiroptera* represents approximately 20% of the known species of mammals [1]. Bats present particular adaptations and strategies among this class, such as hibernation ability, gregariousness, potential for long-distance scattering (the only mammal order capable of flying), long lifespan, and low fecundity [2,3]. In recent years, the conservation status of this taxonomic group has worsened dramatically. As a result, 15% of worldwide bat species are listed as threatened (assessed as critically endangered, endangered, or vulnerable on the Red List of the International Union for the Conservation of Nature) [4]. The origin of their decline is believed to be multifactorial, including infectious diseases such as the white-nose syndrome, which has proven to be a major threat to several bat species in North America [5,6]. However, the existing research efforts in infectious diseases on bats are still focused on the zoonotic potential of pathogens, often neglecting microorganisms as a risk factor to the health and survival of bat populations [6]. This situation highlights the need for studies oriented to infectious diseases affecting bat populations.

*Toxoplasma gondii* is an obligate intracellular protozoan parasite that can infect a wide variety of wild and domestic warm-blooded animals [7]. Its life cycle includes the feline species as the definitive hosts (DH) and all warm-blooded animals, including humans, as the intermediate hosts (IH) [7]. The infection is often asymptomatic in healthy individuals; however, it can be fatal in young, immunocompromised, or congenitally infected individuals [8,9]. In the event of maternal infection during pregnancy, a systemic disease may cause abortion or congenital disease [10]. The only report of clinical toxoplasmosis in bat species occurred in captive flying foxes in Australia, suggesting the food or other fomites originated from the caregivers as the potential spreading route of *Toxoplasma* oocysts into the captive bat colony [9]. Given the increased urbanization and encroachment into natural ecosystems, spread of *Toxoplasma gondii* through human and domestic animal-contaminated sources is likely to become more frequent in wild settings. This may provide new opportunities for the parasite to infect species that had a previous limited contact with it, thus posing a risk to their health. 

In Europe, exposure to *T. gondii* has been extensively described in wild fauna [7]. Particularly, in the Iberian Peninsula, seroprevalences of up to 18.6% have been reported in wild ungulates, 78.8% in carnivores, 68.1% in birds and 17.9% in cetaceans [11,12,13]. However, there is a lack of data regarding the occurrence of *T. gondii* infection in European bats. The few existing studies confirming its presence date back to the 1970s in *Myotis* spp. from France [7], and more recently, to the study by Dodd et al. [14], which reported the presence of *T. gondii* in *Pipistrellus* species from the UK. Studies concerning *T. gondii* infection in the order *Chiroptera* worldwide indicate a wide geographic distribution of *T. gondii* genotypes, as well as a wide range of prevalences of infection depending on the test used, the species and the geographic area of study [15]. These results have led some authors to conclude that bats play an important role in the transmission of *T. gondii* [16]. However, the risk factors, the routes of infection, and the impact of *T. gondii* on bat populations’ health are yet to be fully elucidated. The objective of the present study was to assess the prevalence of *T. gondii* in tissues of pipistrelle bat species from areas densely populated with humans as well as feral cats in NE Spain, in order to broaden the knowledge in the epidemiology of the parasite and the putative role of insectivorous bats as intermediary hosts.

## 2. Materials and Methods

### 2.1. Animals and Samples

**Figure 1:**
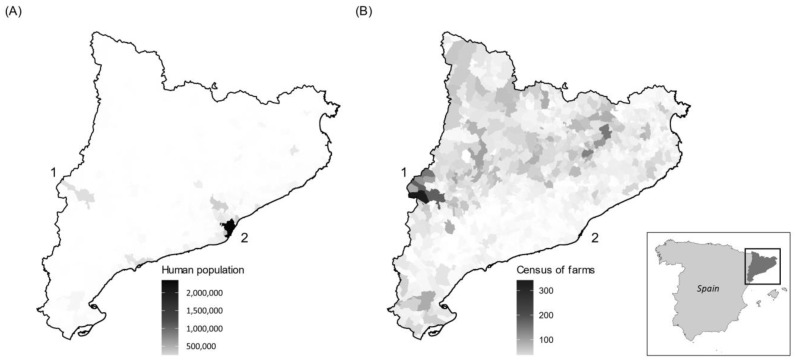
Bat carcasses analyzed in this study were collected from areas 1 (n = 51) and 2 (n = 82) from NE Spain. A gradient of human population is depicted in panel (**A**). Panel (**B**) shows the census of livestock (cattle, sheep, goats and pigs). Area 2 encloses localities with the largest human population of NE Spain (Metropolitan Area of Barcelona) (Panel (**A**)), whereas area 1 encloses localities with the largest abundance of livestock farms of NE Spain (Segrià county, Lleida) (Panel (**B**)).

A total of 133 bat carcasses were supplied by the wildlife rescue centers of Torreferrussa (Barcelona, Spain) and Vallcalent (Lleida, Spain). Bats were originally found dead, died during their stay in the rescue center without relevant clinical signs, or were euthanized for welfare reasons (e.g., severe traumatic injuries). Animals of this study were collected from the most densely human-populated area of NE Spain [17] (n = 82) and from an area with a high density of livestock farms (n = 51), over a three-year period (2019–2021) (Figure 1). Data on the density of farms (cattle, sheep, goats and pigs) was provided by the Departament d’Acció Climàtica, Alimentació i Agenda Rural (i.e., Department of Climate Action, Food and Rural Agenda) of the Government of Catalonia. Both factors, human and farm densities, have been associated with a high abundance of cats [18] and a high environmental contamination with *T. gondii* oocysts [19]. 

The three sympatric species of *Pipistrellus* bats present in the territory were *P. pygmaeus* (n = 70), *P. kuhlii* (n = 42) and *P. pipistrellus* (n = 21). Within the sampled animals, there was a total of 67 females (50.4%) and 66 males (49.6%), of which 68 were adults (51.1%), 51 juveniles (38.4%) and 14 newborns (10.5%). These three sympatric *Pipistrellus* species are small crevice-dwelling bats adapted to thrive in urban areas. *P. pipistrellus* may be sedentary or regional migrants depending on the area. Males are solitary most of the year, while breeding females congregate in colonies ranging from a few to a thousand individuals [20]. There is hardly any information on seasonal movements in *P. pygmaeus*. Their breeding colonies tend to be more numerous than those of *P. pipistrellus*, although they may alternate between several different shelters during this period [21]. *Pipistrellus kuhlii* is a sedentary species that can use the same shelters throughout the year. During the breeding season, females form colonies ranging from 15 to 50 [22]. All three species are strict insectivores, although there are slight differences in the types of insects they predate; for example, *P. pygmaeus* has a less developed mandible that forces it to eat smaller prey [21].

All bat carcasses were examined under Level-2 biosafety measures in the laboratory (CReSA-IRTA, Bellaterra, Spain), in a vertical laminar air flow (LAF) cabinet with HEPA filter and while wearing personal protection equipment (PPE). At least one of the following samples was collected per carcass: central nervous system (n = 132), myocardium (n = 115), fetus (n = 2) and uterus (n = 1). All collected samples were stored at –80 °C until analysis.

### 2.2. Molecular Analysis

Samples (0.1 g tissue/sample) were individually homogenized with 475 µL of sterile phosphate-buffered saline (PBS) using the TissueLyser II (QIAGEN, Leipzig, Germany) for 10 min at 30 Hz. After this, they were mixed with 25 μL of proteinase K and consecutively vortexed and incubated at 56 °C until complete lysis was obtained (between 1 and 3 h for central nervous system, and overnight for myocardium, fetus and uterus samples). DNA was extracted from samples using the commercial kit Indimag Pathogen Kit (INDICAL biosciences GmbH, Leipzig, Germany), following manufacturer’s instructions. The extracted DNA was amplified performing a qPCR using the primers Toxo-SE (5′ AGGCGAGGGTGAGGATGA 3′) and Toxo -AS (5′ TCGTCTCGTCTGGATCGCAT 3′), the probe (5′ 6FAM— CGACGAGAGTCGGAGAGGGAGAAGATGT—BHQ1 3′) and the commercial kit TaqMan™ PCR Master Mix (Applied Biosystems™, Carlsbad, CA, USA) [23]. Primers Toxo-SE and Toxo-SA target the 529 bp repeat region (REP529, GenBank accession no. AF146527) of *T. gondii* [23]. The qPCR method used is able to detect *T. gondii* DNA extracted from a single cyst DNA [23,24]. *Toxoplasma gondii* oocysts (purchased at *Grupo SALUVET, Departamento de Sanidad Animal, Facultad de Veterinaria, Universidad Complutense de Madrid, Spain*) were used as positive control. Each qPCR run included a negative control, containing 500 µL of PBS. The cycling protocol was as follows: initial decontamination at 50 °C for 2 min and denaturation at 95 °C for 10 min, followed by 40 cycles at 95 °C for 15 s and 61 °C for 1 min.

### 2.3. Statistical Analyses

A convenience sample of 133 bat carcasses was tested for the presence of *T. gondii.* That allowed for the detection of 2.2% prevalence with a 95% confidence level, as calculated by the following formula (derived from the approximate formula for sample size calculation for an infinite population; see [25] for details):

$$p=1-\frac{ln\alpha }{n}$$

where *n* is the required sample size, α is one minus the confidence level and *p* is the prevalence of detection.

## 3. Results

All examined tissues tested negative for the presence of *T. gondii* (0.0% prevalence with 95% CI: [0.0–2.6]). A Fisher’s exact test showed a significant difference (*p* = 0.0002) between the prevalence in our study and the prevalence in the study of Dodd et al. [14].

## 4. Discussion

The present study concludes that *Pipistrellus* spp. bats do not seem to play a significant role in the epidemiology of *T. gondii* in NE Spain, in contrast to other sympatric wildlife species [11,12,13,26]. The absence of this parasite in *Pipistrellus* spp. from our study concurs with what would be expected considering their feeding behavior, since they are strictly insectivorous species [21]. In contrast, previous serological and molecular studies have evidenced high rates of *T. gondii* infection in insectivorous bats [15] and multiple risk factors, other than trophic sources, have been proposed: different ecological traits of bat species (gregariousness, dietary habits), environmental conditions and density of oocysts in the environment [1,14,16,27,28]. The present is the third study focused on the presence of *T. gondii* in insectivorous bats performed in Europe. Unlike the previous studies [15], our result suggests that *T. gondii* infection is not so widely prevalent in insectivorous bats from NE Spain. Particularly, there is a statistically significant difference compared to the results obtained by Dodd et al. [14], who found 10.39% positivity of *T. gondii* DNA in *P. pipistrellus* and *P. pygmaeus* in the UK. Animals analyzed in both studies are believed to be exposed to a comparably high cat population density [29,30], a factor with an integral influence on the number of oocysts in the environment [31]. Sporulated oocysts may remain viable for years under humid conditions, resisting heat and cold temperatures. However, immature oocysts’ survival is threatened by temperatures under –6 °C or above 20 °C, where critical dissection may occur [32]. Therefore, the same environmental exposition of insectivorous bats to *T. gondii* in Spain and the UK cannot be granted since meteorological variations between both geographic areas are expected to determine the survival and spread of oocysts [33].

High seroprevalences of *T. gondii* infection have been reported in wildlife species from the UK and NE Spain [13,34], confirming that wildlife is highly exposed to the parasite in both geographic areas. Three routes of infection with *T. gondii* have been reported: 1) fecal–oral, through the ingestion of oocysts shed by the DH into the environment; 2) ingestion of tissue cysts (bradyzoites) through consumption of raw or undercooked infected meat of IHs; and 3) vertically, through transplacental transmission of tachyzoites from mother to fetus in both the IH and the DH [7]. The route of infection of *T. gondii* in bat populations is far from being understood. While drinking from water sources contaminated with oocysts may be a plausible explanation for all species, their specific dietary habits (consumption of fruit and pollen, insects, blood or vertebrates) may provide unique and characteristic infection pathways [35]. Due to their feeding behavior and almost exclusively aerial life, the horizontal transmission in insectivorous bats seems unlikely. Besides drinking from oocyst-contaminated water, infection is believed to occur by consuming insects acting as mechanical vectors [35,36,37]. Although the congenital transmission is not the main route of persistence of *T. gondii* in mammals [7], further research is needed to understand the significance of this transmission in the maintenance and spread of the parasite in microchiropteran species. In Dodd et al. [14], different factors made it impossible to allow the exploration of congenital transmission in the population of bats. However, through genotyping studies, they observed that most of their examined bats derived from the same interbreeding population [14]. This result suggests that once infected, vertical transmission may play an important role in the maintenance and amplification of the parasite in isolated bat populations [1,14,15,27]. The existence of an independent sylvatic *T. gondii* cycle in bats has been discarded, as genotyping studies have revealed that bats share the same isolates found in domestic and wild terrestrial animals [16,27,28,36].

Contrary to most mammal species, marsupials and New World primates manifest severe and fatal clinical presentations [38,39]. In bat populations, the determinants of mortality and disease have been historically difficult to identify, partly due to the fast decomposition of carcasses just after death and the challenges encountered to access the locations where bats dwell [40]. Accordingly, the potential detrimental effect of *T. gondii* on insectivorous bats living under certain ecological conditions could go largely unnoticed. Our results should incentivize larger-scale research and extend its coverage beyond wildlife rehabilitation centers, where reasons for admission usually are biased towards traumatism and opportunistic encounters [40,41].

## 5. Conclusions

*Pipistrellus* spp. appear not to play a significant role in the epidemiology of *T. gondii* in NE Spain. Further studies are encouraged to elucidate both the epidemiology of *T. gondii* and its potential impact on the health of microchiropteran species in Europe. 

## Data Availability

Not applicable.
